# Raman spectral signature reflects transcriptomic features of antibiotic resistance in *Escherichia coli*

**DOI:** 10.1038/s42003-018-0093-8

**Published:** 2018-07-02

**Authors:** Arno Germond, Taro Ichimura, Takaaki Horinouchi, Hideaki Fujita, Chikara Furusawa, Tomonobu M. Watanabe

**Affiliations:** 1 RIKEN Center for Biosystems Dynamics Research, 6-2-3 Furuedai, Suita, Osaka 565-0874 Japan; 2 Department of Transdimensional Life Imaging, Open and Transdisciplinary Research Initiatives, Osaka University, 1-1 Yamadaoka, Suita, Osaka 565-0871 Japan; 3 Waseda Bioscience Research Institute in Singapore (WABIOS), 11 Biopolis Way, #05-02, Helios, Singapore, 138667 Singapore; 4 Universal Biology Institute, The University of Tokyo, 7-3-1 Hongo, Tokyo, 113-0033 Japan

## Abstract

To be able to predict antibiotic resistance in bacteria from fast label-free microscopic observations would benefit a broad range of applications in the biological and biomedical fields. Here, we demonstrate the utility of label-free Raman spectroscopy in monitoring the type of resistance and the mode of action of acquired resistance in a bacterial population of *Escherichia coli*, in the absence of antibiotics. Our findings are reproducible. Moreover, we identified spectral regions that best predicted the modes of action and explored whether the Raman signatures could be linked to the genetic basis of acquired resistance. Spectral peak intensities significantly correlated (False Discovery Rate, *p* < 0.05) with the gene expression of some genes contributing to antibiotic resistance genes. These results suggest that the acquisition of antibiotic resistance leads to broad metabolic effects reflected through Raman spectral signatures and gene expression changes, hinting at a possible relation between these two layers of complementary information.

## Abstract (web summary)

Techniques for characterizing the mode of action of antibiotic resistance are crucial for developing new antimicrobial drugs. Arno Germond et al. have used Raman spectroscopy combined with gene expression to investigate large metabolic changes that occur when bacteria acquire antibiotic resistance.

## Introduction

The acquisition of antibiotic resistance in bacteria raises important concerns for public health. While the number of pathogens resistant to various drugs is on the rise, various scientific and economic challenges have reduced the development of new antibiotic drugs^1, 2^. It has been suggested that the development of antibiotic drugs could be accelerated by techniques that could perform rapid and accurate characterization of the mode of action, or mechanism, of bacterial resistance. Such approaches would also be beneficial in clinical environments, where patient survival depends on the time needed to diagnose patients infected by antibiotic-resistant bacteria, which needs to be shortened as much as possible. However, the conventional methods used to detect antibiotic resistance, such as cell culture, in vitro drug sensitivity testing, proteomic profiling, and genetic analyses (e.g., transcriptomic analyses), are time-consuming and costly.

Raman spectroscopy is increasingly used in microbiology to perform cell-type or cell-state identification, with the possibility of achieving single-cell measurements^3, 4^. Because a Raman spectrum is essentially an ensemble of molecular vibrations, it provides rich, but complex information reflecting the metabolism and chemical composition of the cell and its structures. Raman spectroscopy has been successfully used to monitor the phenotypic responses of bacterial species exposed to various drugs, at various concentrations, and for various durations of exposure^5–17^. Cost-efficient and fast identification measurements of various types and subtypes of pathogenic and commensal bacteria and mycobacteria exposed to drugs were reported^14, 16, 17^, demonstrating the potential of this technique by comparison to more conventional approaches. In addition, some studies also hinted at possible metabolic or structural relations between cells and spectral signatures^15, 17, 18^.

The question remains whether spectral information can be used to reliably discriminate acquired resistance in bacteria in the absence of drugs, and if the spectral signatures can be linked to the molecular mechanisms of antibiotic resistance. These two questions are challenging because of our limited understanding of the complex nature of the Raman spectrum, in which each peak represents the contribution from multiple chemical compounds. In order to increase the interpretability of the Raman spectrum phenotypic signature, we envision it could be combined with the information of other techniques. Genetic analyses of microbial resistance against antibiotic drugs have been a topic of high interest in recent years, and some specific genes were contributing to the antibiotic-resistance phenotypes have been identified^19, 20^. Since Raman spectral data have been shown to be good phenotypic indicators of antibiotic resistance, it is worth exploring if it reflects variations in the gene expression of *Escherichia coli* strains. It is reasonable to assume that information derived from two different facets of a common biological system may be linked through the complex network of the cell.

In this paper, we explore these questions using antibiotic-resistant *E. coli* MDS42 cells obtained through laboratory evolution^20^. We used ten strains that exhibit mutations conferring resistance to different antibiotics (Table 1). Specifically, strains CFIX and CPZ are resistant to β-lactam antibiotics, which inhibit cell wall synthesis; strains CPFX and ENX are resistant to quinolones, antibiotics that act on DNA gyrase systems and that participate in DNA replication; and strain TP is resistant to trimethoprim, which inhibits DNA replication by preventing the synthesis of folic acid, a precursor to the essential coenzyme tetrahydrofolate. We also considered five strains that show resistance to antibiotics that inhibit the translation of mRNA by binding to the 30S ribosomal subunit or the 50S ribosomal subunit, and thereby prevent the synthesis of proteins.

**Table 1:** List of strains used in this study

Evolved strains	Antibiotic name	Class	Mode of action	Representative fixed mutations
CFIX	Cefixime	Cephalosporin, β-lactam	Cell wall	*ompC*
CPZ	Cefoperazone	Cephalosporin, β-lactam	Cell wall	*acrB*, *acrR*
CPFX	Ciprofloxacin	Quinolone	DNA gyrase	*gyrA*, *ompF*, *mipA*, *nuoA*
ENX	Enoxacin	Quinolone	DNA gyrase	*gyrA*, *acrR*, *dinG*
TP	Trimethoprim		Folic acid synthesis	*phoQ*
AMK	Amikacin	Aminoglycoside	Protein synthesis 30S, aminoglycosides	*cpxA*, *phoQ*, *nuoE*
DOXY	Doxycycline	Tetracycline	Protein synthesis 30S, aminoglycosides	*rpsF*
NM	Neomycin	Aminoglycoside	Protein synthesis 30S, aminoglycosides	*cpxA*, *cyoAB*, *ompC*, *sapA*
AZM	Azithromycin	Azalide, macrolide	Protein synthesis 50S	*gyrA*
CP	Chloramphenicol		Protein synthesis 50S	*acrR*, *marR*, *mutL*, *ompR*

The name of the strains corresponds to the antibiotics they were exposed to during a 3-month period of experimental evolution after which it was confirmed that the resistance phenotype was maintained in the absence of antibiotics^20^. Genomes of each strain were sequenced in a previous study^20^ and fixed mutations found in the evolved strains are listed

First, we determined whether Raman spectra could discriminate the different strains that developed resistance. We evaluated the robustness and reproducibility of the discrimination by analyzing a relatively large number of independent bacterial population. We then investigated the possibility of extracting the wavenumbers of Raman spectra that contributed to the discrimination. The hope was that these same wavenumbers could then be linked to the mode of action for each form of antibiotic resistance. Our results show that Raman spectroscopy identifies both the type of antibiotic resistance and the mode of action across the 11 considered strains in a reproducible manner. In addition, we explored the relationship between the expression of some well-known antibiotic resistance genes, and the Raman spectral intensities. Significant linear correlations (|*R*| > 0.601, FDR *p* < 0.05) were found, suggesting that the expression of multiple genes induces detectable spectroscopic variations. Our results strongly encourage further studies to verify the possibility of predicting antibiotic resistance in other species in the absence of drugs, and demonstrating how gene expression and spectral data might be connected through the network of the cell by integrative machine learning techniques.

## Results

### Discrimination of 11 bacterial strains

**Fig. 1:**
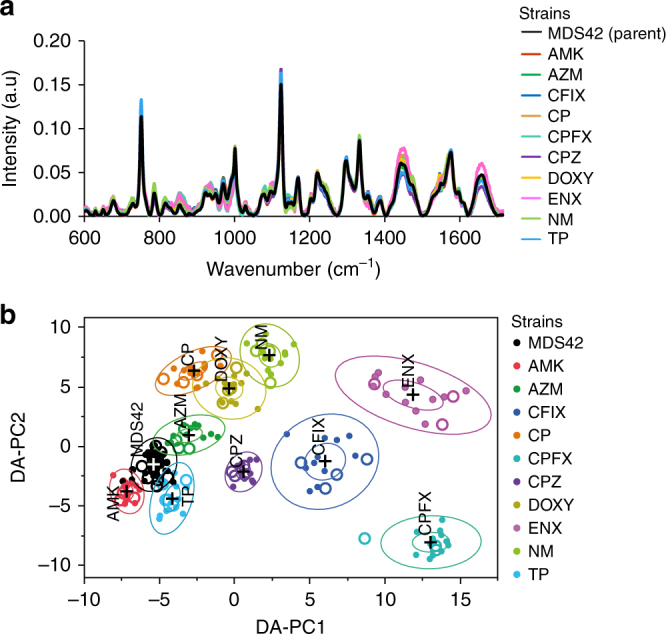
Label-free spectral measurements of 11 *E. coli* strains and discrimination of the strains. **a** Normalized mean Raman spectrum of the *E. coli* parental strain MDS42 (*n* = 48 independent population) and each of the laboratory-evolved antibiotic-resistant strains (*n* = 16 independent cell cultures). **b** Discrimination of *E. coli* cell type on the first two DA–PC dimensions of the DA–PC model. Inner and outer rings represent 95% and 50% confidence-levels, respectively. The model was trained using 155 population (filled circles), then 52 population chosen randomly from each cell line were used as test data (white circles) and were plotted on the same semantic space. Bacterial population of the test data were classified by type with 100% accuracy

The parental *E. coli* strain and ten antibiotic-resistant laboratory-evolved strains obtained from a previous study^20^ were grown in the absence of antibiotics using a robotic system. Immediately following culturing, strains were measured by Raman spectroscopy using optical-bottom 96-well plates (see Material and Methods). Each well contained an independently grown cell culture (i.e., biological replicate), and the bacterial cells were measured at five different locations within the well. After appropriate background subtraction and normalization, the spectroscopic profiles of the bacterial cells were averaged to obtain a spectrum representative of a given bacterial population (single well). For all subsequent analysis, we consider these single well population as representative units. The normalized mean spectra for the parental strain derived from 48 individual wells and for the ten evolved strains each derived from 16 individual wells are shown in Fig. 1a.

Differences in intensities were clearly visible among the 11 strains (*n* = 208 populations). A total of 35 peaks regions were selected from literature and visual inspection of the average spectrum of each cell line, then associated to molecular compounds (Table 2). To demonstrate the statistical significance of the variation across the strains, ANOVA *F*-values and Fisher scores, two methods that estimate variance, were calculated using the normalized spectra (Supplementary Figure 1). The differences in spectral intensities were significant (ANOVA, *p* < 0.01) for all wavenumbers corresponding to peak regions identified in Table 2; however, intensity differences for some of the valley regions corresponding to the baseline of the Raman spectra were not statistically significant. The spectral shapes of the ANOVA *F*-value and Fisher score were similar to the average spectrum of each strain, and that weak peaks made large contributions to the discrimination of the strains (Supplementary Figure 1). For example, four peaks in the 800–1000 cm^−1^ spectral region (853, 936, 972, 989 cm^−1^) were weakly observed, but highly contributed to the discrimination. These spectroscopic vibrations were primarily related to the skeletal structure of proteins (Table 2). The relationship between some of these peaks and particular genes will be discussed in a later section.

**Table 2:** Molecular assignment of the Raman peaks found in this study.

**Wavenumber (cm** ^**−1**^ **)**	**Molecular assignment**		**References**
~605		Cholesterol	Surmacki et al.^29^
~630	δ(C–C) twist., Tyr	Protein	Notingher et al.^30^ Teng et al.^15^
~650	C–S stretch., C–C twist.	Protein	Teng et al.^15^
~676	G, T, C–S stretch. of cysteine	Nucleic acid, protein	Notingher et al.^30^ Teng et al.^15^
~730	A ring breath.	Nucleic acid	Notingher et al.^30^
~752	δ(C–C) Tyr	Protein, cytochrome	Notingher et al.^30^
~786	C, T, U ring br., ν PO_2_ group	Nucleic acid	Notingher et al.^30^ Moritz et al.^9^
~818	O–P–O stretch. DNA, Tyr	Nucleic acid, protein	Teng et al.^15^
~853	ν(C–C) proline, ring breath. Tyr	Protein (glycogen, collagen)	De Gelder et al.^31^ Notingher et al.^30^
~878	ν(C–C), COH ring	Lipid, carbohydrate	Notingher et al.^30^
~922	R-CH_3_	L-alanine	De Gelder et al.^31^
~936	C–O–C linkage, C–C stretch., α-helix	Carbohydrate, protein	De Gelder et al.^31^
~950		Cholesterol	Teng et al.^15^ Surmacki et al.^29^
~972	CH_2_ rock., C–C stretch., α-helix	Protein, lipid	Moritz et al.^9^
~989	β-sheet	Protein, histamine	De Gelder et al.^31^
~1001	Phe ring breath., C–C skeletal (protein)	Phenylalanine, protein	Notingher et al.^30^ Teng et al.^15^
~1030	δ(CH) bend., Tyr, Phe	Aromatic compound	De Gelder et al.^31^ Notingher et al^30^.
~1079	PO2 str., (C–C) stretch., C–O	Nucleic acid, lipid, carbohydrates	Notingher et al.^30^
~1101	Symmetric phosphate stretch. (DNA)	Nucleic acid	Teng et al.^15^
~1123	CH Phe	Cytochrome	Notingher et al.^30^ Teng et al.^15^
~1155	CC/CN stretch.	Protein	Notingher et al.^30^ Teng et al.^15^
~1170	C–H in-plane bend. mode (Tyr), (CH) Phe	Protein	Teng et al.^15^
~1209	C–C_6_H_5_ stretch., Phe, Trp	Protein	Notingher et al.^30^
~1220-40	T, A, Amide III, CH bend.	Nucleic acid, protein, lipid	Notingher et al.^30^
~1298	CH_2_ twist.	Saturated lipid	Notingher et al.^30^
~1333	CH_3_CH_2_ def. of collagen	Nucleic acid, protein	Teng et al.^15^
~1355	A, G, CH def.	Nucleic acid, protein	Notingher et al.^30^
~1388	CH_3_	Lipid	Teng et al.^15^
~1450	G, A, CH def.	Nucleic acid, protein, lipid, carbohydrate	Notingher et al.^30^
~1476	Amide II, Purine bases (U)	Cytochrome *bo*, nucleic acid	Moritz et al.^9^ Teng et al.^15^
~1545	υ(C=C) stretch., Tyr	Protein	Moritz et al.^9^
~1578	G, A	Nucleic acid	Notingher et al.^30^ Teng et al.^15^
~1599	υ(C=C) aromatic compound	Phenylalanine, tyrosine	Notingher et al.^30^ Teng et al.^15^
~1610	υ(C=C), Trp	Protein	Notingher et al.^30^ Teng et al.^15^
~1658	υ(C=C) cis., amide I envelope	Unsaturated fatty acid, lipid, protein	Notingher et al.^30^ Teng et al.^15^

*stretch.* stretching mode, *bend.* bending, *br.* breathing mode, *def.* deformation, *twist.* twisted, *Tyr* tyrosine, *Trp* tryptophan, *Phe* phenylalanine

In Fig. 1b, we applied principal component analysis (PCA) followed by discriminant analysis, a supervised approach we refer to as DA–PC, which allowed us to quantitatively represent each bacterial population in a space of reduced dimensions. The PCA results are described in detail in Supplementary Figure 2. First, we tested if the spectral variations observed in Fig. 1a allowed for the discrimination of the cells by cell-type, despite their close relationship. Spectral dataset was separated into training and test dataset so that 25% of the dataset is used as an independent test dataset. Specifically, we randomly selected four biological replicates per evolved strain, and 12 for the ancestral strain for the test data. Eight principal components were selected in the model based on their Fisher score (see Materials and methods). The DA–PC model was performed with the training data. The 95% and 50% confidence-level ellipses are plotted for the mean of each strain. When groups differed significantly, their confidence ellipses tend to not intersect. The DA–PC model exhibited 100% well-classified observations on the training dataset (filled markers), and successfully discriminated the test data (white markers) with an accuracy of 100% (Fig. 1b). The result suggests that Raman spectroscopy has the ability to discriminate and predict the 11 types of bacteria strains, even though these strains are genetically very close. A similar result was obtained when using a larger dataset from three independent experiments, as described later.

### Discrimination of the mode of action of acquired antibiotic resistance

**Fig. 2:**
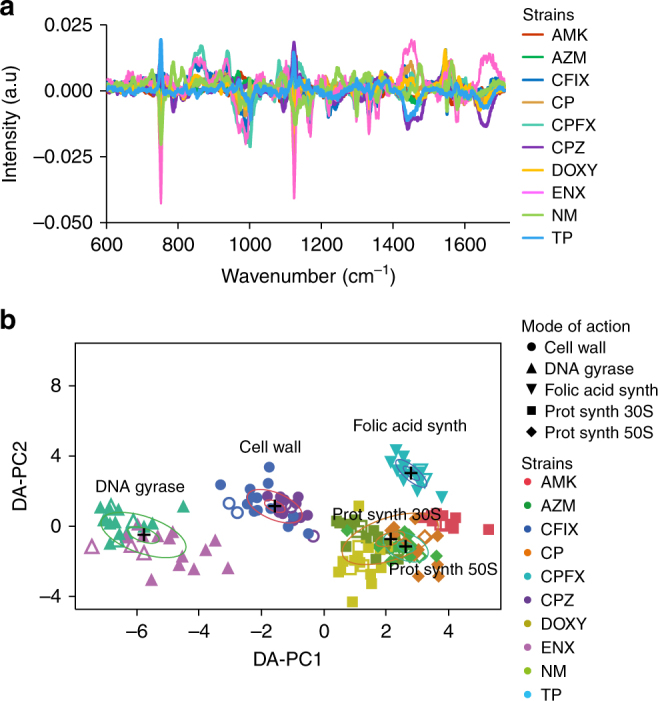
Discrimination of the mode of action of antibiotic resistance in absence of antibiotics. **a** Relative spectrum differences of each population of the laboratory-evolved antibiotic-resistant strains from the averaged spectrum of the parental population (*n* = 48). **b** Discrimination of the mode of antibiotic resistance on the first two DA–PC dimensions of the DA–PC model. The DA–PC was performed on the spectral differences shown in (**a**). The model trained on 120 population (filled markers) and 40 population chosen randomly from each cell line were used as test data (white markers). Inner and outer rings represent 95% and 50% confidence-levels, respectively. Bacterial population of the test data was classified according to their respective mode of action of antibiotic resistance with a 99.4% accuracy

We hypothesized that the effects of acquired resistance would be reflected in metabolic and/or structural differences between the parental and evolved strains. Thus, we calculated the differences between the spectra of the evolved strains (*n* = 16 per strain) and the averaged spectrum of the parental strain (*n* = 48) (Fig. 2a). The relative differences between the parental and evolved strains are shown for a few selected peaks, as well as across the entire spectral range (Supplementary Figure 3a and 3b, respectively). Notably, important differences across strains were found for nucleic-acids, protein-related and/or cytochrome-related aromatic compounds, proteins, aromatic compounds, and lipids. To determine if the relative differences across the ten evolved strains were statistically significant, we calculated the ANOVA *F*-value and Fisher scores on the difference spectra (Supplementary Figure 4). Variations of relative differences were found to be significant (ANOVA, *p* < 0.001) for all the peak regions.

We then investigated whether the relative spectral differences (Fig. 2a) reflected the five mechanisms of antibiotic resistance. A DA–PC model was developed using the relative spectral differences for 160 population. Four biological replicates per evolved strain were randomly selected to constitute a test data. Seven principal components of the PCA model were selected according to their *F*-values (Supplementary Figure 5). DA–PC model was trained and cross-validated using the training data, and exhibited 100% well-classified observations on training data. When using the test data, only one population was found misclassified. The overall prediction accuracy was 99.4%, with an *R*^2^ of 0.98. In the DA–PC model shown in Fig. 2b, the evolved strains clustered together, depending on the mode of action of their antibiotic resistance. For example, clones CFIX and CPZ were grown during experimental evolution with cefoperazone and cefixime stresses. Both of these antibiotics target the cell wall. When grown in the absence of antibiotic, these two strains clustered in the DA–PC analysis. Likewise, strains CPFX and ENX (DNA gyrase), strains AMK, DOXY, and NM (protein synthesis, 30S rRNA), and strains AZM and CP (protein synthesis, 50S rRNA) grouped according to the specific mode of action of their antibiotic resistance. The overlap observed between the groups whose resistance mechanisms are both through protein synthesis (30S and 50S) suggests that their difference was not statistically significant when only two DA–PC dimensions were considered. The above results suggest that Raman spectroscopy has the ability to discriminate and predict the four major modes of action of antibiotic resistance, which was confirmed when considering a larger dataset obtained from three independent experiments, as described below.

### Reproducibility and outcome predictability of independent experiments

Because biological and technical variations, such as temperature and vibrational effects on optical components, are known to influence the results of Raman spectroscopy, this technique is often criticized for its lack of reproducibility^21^. Reproducibility can be defined as the limitation in discriminating different cell-lines across experiments that were performed at different times. Good reproducibility can be defined as the ability to successfully predict the result of an independent experiment. In the present study, we made a point to demonstrate the reproducibility of our results. To accomplish this, three independent spectral measurements of 208 independent cell-cultures were performed during different weeks. The datasets were combined and evaluated by subsequent multivariate analyses (Supplementary Figure 6). The first eight statistically significant PC components (*p* < 0.001) were chosen for subsequent discriminant analyses. DA–PC showed that each strain occupied a similar position in the semantic space across three experiments (Supplementary Figure 6c, 6d, 6e). While some variations were observed across experiments, the strains were classified with an accuracy of 99.6%. This result demonstrates the robustness of the classification across independent experiments, performed at different times.

To evaluate the reproducibility of our method, we trained a predictive DA–PC model across two combined datasets of spectral measurements obtained on different days (training data, *n* = 416), and tested it against a third dataset obtained on a different day (test data, *n* = 208). Cross-validation within the training data exhibited 97.9% well-classified observations, with an *R*^2^ of 0.96. When tested against the test data, a classification accuracy of 93.3% was achieved (14 misclassified spectra out of 208) and the predicted *R*^2^ value was 0.81. These results verified that our experimental approach could predict the identity of bacteria measured in an independent experiment.

Likewise, we tested whether the mode of action of antibiotic resistance could be predicted. We used the same datasets as above. However, in this test, the difference spectra were calculated by subtraction of the mean spectrum of the parental strain from each mode of action from two datasets that were concatenated (training data, *n* = 320), and tested against the test dataset (*n* = 160) obtained from the third experiment. The DA–PC model included seven PCs and cross-validation of the training data accurately classified 95.3% of the observations, with an *R*^2^ of 0.88. When tested against the test dataset, 93.7% of the 160 bacterial cultures were successfully classified according to their mode of action for antibiotic resistance in the absence of antibiotics. The predicted *R*^2^ value was 0.86, demonstrating a good ability to identify the mode of action of antibiotic resistance from a new dataset.

### Spectral peaks important for the discrimination antibiotic resistance mode of action

**Fig. 3:**
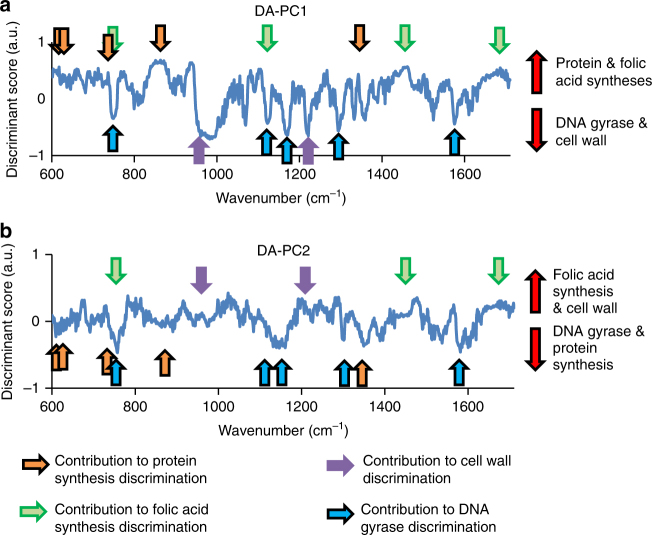
Vector shapes of canonical discriminant analysis axes of the first (**a**) and second dimensions (**b**) of the DA–PC model. Arrows show the contribution of spectral peaks to the various mode of action of antibiotic resistance. The position of the arrows corresponds to the maximum value of some of the major spectral peaks

In an attempt to extract the wavenumbers that contributed to the discrimination of each mode of action (Fig. 2b), we established a DA–PC model that included all population as training data, then extracted the discriminant vectors (vectors of the canonical discriminant analysis axes) of the first two components, as shown in Fig. 3. The positive values of DA–PC1 contributed to the modes of action of protein synthesis, 30S and 50S, and folic acid synthesis; while negative values primarily contributed to the DNA gyrase group. In the DA–PC2, the positive values contributed to the discrimination of the folic acid synthesis and the cell-wall associated modes of action. Negative values of DA–PC2 contributed to the classification of the DNA gyrase group and the protein synthesis group, although these groups could also exhibit positives values in the two-dimensional space of the DA–PC model (Fig. 2b). Together, these results show that the whole spectral region (600–1710 cm^−1^) successfully identified the different modes of action of antibiotic resistance, but that some spectral regions could contribute toward a given mechanism of action.

The peaks at wavenumbers ~950 and ~1209 cm^−1^ exhibited negative values in the DA–PC1, and positive values in the DA–PC2. We considered these peaks as contributing to the discrimination of the cell wall-related group in the DA–PC model, although the associated discriminant scores at these wavenumbers are not that dominant (Fig. 3). The peaks at ~752 and ~1123 cm^−1^, often assigned to cytochrome (Table 2), and the peaks at ~1170 cm^−1^ (proteins), ~1298 cm^−1^ (saturated lipids), and ~1578 cm^−1^ (nucleic acids), were identified in both the discriminant vectors with negative values. These peaks were considered to contribute to the classification of the DNA gyrase group. The peaks at ~605, ~630, ~730, ~853, and ~1350 cm^−1^ exhibited positive values in the DA–PC1, and negative values in the DA–PC2, and were considered to contribute in the discrimination of the protein synthesis group. The peaks at ~1450, ~1545, and ~1658 cm^−1^ were attributed to the folic acid synthesis group for both of the discriminant vectors. The peaks at ~752 and ~1123 cm^−1^ also contributed negatively to the classification of the folic acid synthesis group.

### Correlations between peak spectral intensities and gene expression

**Fig. 4:**
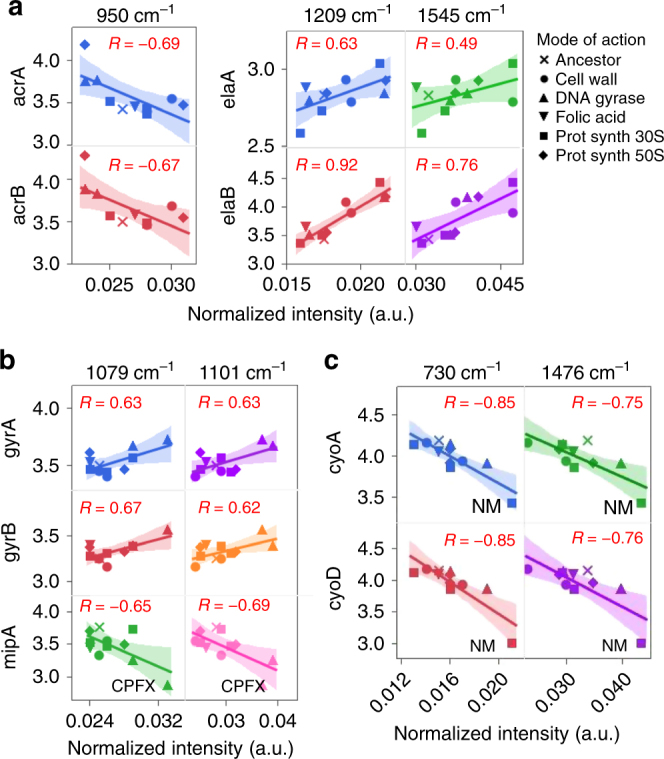
Scatterplots of normalized Raman peak intensity and normalized gene expression for genes related to three modes of action, cell wall (**a**), DNA gyrase (**b**), and protein synthesis (**c**). Wavelengths that helped identify these modes of actions were selected and associated to genes that may contribute to these antibiotic resistances. On each scatter plot, each point represents a strain for which the gene expression was measured by microarray, and the spectral intensities were averaged from 16 population (laboratory-evolved strains) or 48 population (parental strain). A linear fit was applied to each scatterplot, and the Pearson correlation value *R* is displayed on each graph. Two-tailed test and FDR assessed that correlations greater than 0.601 in absolute value were significant (|*R*| > 0.601, FDR *p* < 0.05)

**Fig. 5:**
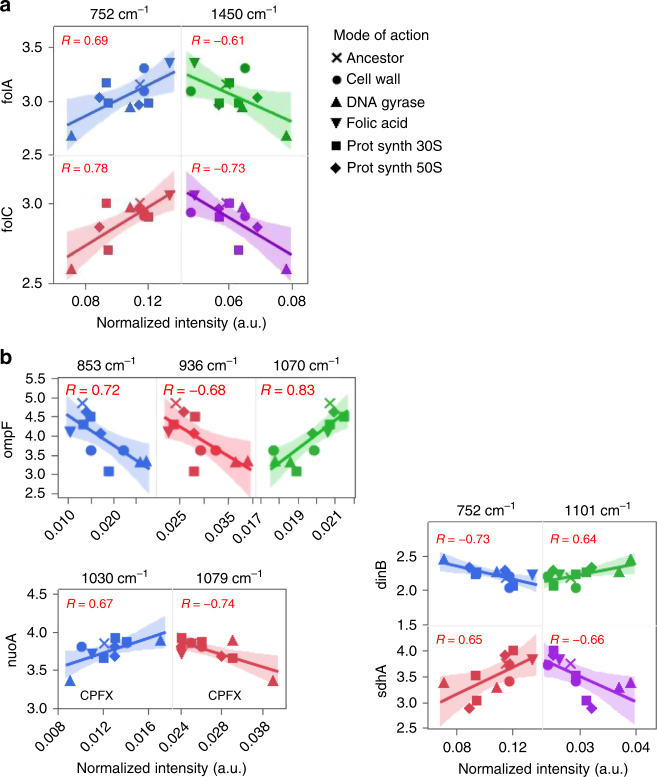
Scatterplots of normalized Raman peak intensity and normalized gene expression for folic acid synthesis (**a**) and other genes of interest (**b**). Wavelengths that helped identify these modes of actions were selected and were associated to genes known to contribute to the mode of action for folic acid synthesis or known to contribute to various antibiotic resistances were selected. A linear fit was applied to each scatterplot, and the Pearson correlation value *R* is displayed on each graph. Two-tailed test and FDR assessed that correlations greater than 0.601 in absolute value were significant (|*R*| > 0.601, FDR *p* < 0.05)

A number of genes contributing in various ways to antibiotic resistance are known^19, 20^, and we identified above various groups of peaks that contribute to discriminate the modes of action of antibiotic resistance. Here we investigated the correlation of the expression some of these genes with the Raman spectra. Gene expression related to antibiotic resistance for the 11 strains were obtained at the population level and are expressed in normalized quantile^20^. Scatter-plots of normalized gene expression of selected genes compared to normalized spectral intensities of selected wavenumbers across the 11 strains are represented in Figs. 4 and 5. Correlations between spectral intensities and gene expression were calculated and considered significant if the absolute value of the correlation coefficient was greater than 0.601 (|*R*| > 0.601, *p* < 0.05, see Materials and methods). We have chosen to showcase some of the positive and/or negative correlations found for some genes of interest (Figs. 4 and  5).

We selected a few genes coding for inner membrane protein and multidrug efflux pump which might contribute in cell-wall resistance (Fig. 4a). The genes *acrA* and *acrB* code for a multidrug efflux pump that organisms use to expel drugs out of their cells, as well as also for cation and protein export^22^. Gene expression of *acrA* and *acrB* correlated significantly (FDR, *p* < 0.05) to the spectral intensity of wavenumber attributed to lipids (~950 cm^−1^) (Fig. 4a), which was identified as being important to the cell wall group (Fig. 3). The wavenumber ~1209 cm^−1^ also correlates with the cell wall cluster (Fig. 3a, b). Interestingly, the expression of *elaA* and *elaB* was correlated with the spectral intensity of this wavenumber (FDR, *p* < 0.05). These two genes encode an inner cell wall membrane protein that are involved in various stress responses^23^. In addition, *elaB* was also correlated to ~1545 cm^−1^ (FDR, *p* < 0.05), but not *elaA*. The above correlations suggest a relation between these wavenumbers and genes. The strains CFIX and CPZ, which were grown under antibiotics targeting the cell-wall (Table 1), did not exhibit higher spectral intensity or gene expression at 1209 cm^−1^. This hints at the contribution of genes other than *elaA* and *elaB* in resistance in these two strains.

Significant correlations (FDR, *p* < 0.05) were found between three genes often involved in DNA gyrase-related antibiotic resistance and the spectral intensities at wavenumbers 1079 and 1101 cm^−1^ (Fig. 4b), both of which are associated to nucleic acids (Table 2). We noticed that for *mipA*, which is a regulator gene of *gyrA* and *gyrB*, correlation coefficient values had opposite signs to the ones for *gyrA* and *gyrB*. Two strains associated with DNA gyrase stress (ENX and CPFX) displayed higher spectral intensities at 1101 cm^−1^, compared to the other strains (Fig. 4b). The difference was found to be significant (ANOVA, Tukey HSD, *p* < 0.05). The CFPX strain, which exhibits a fixed mutation in *mipA* (Table 1), exhibited the lowest level of *mipA* expression in comparison to any other strain (Fig. 4b).

Mutations in the *cyo* operon, which encodes for a cytochrome subunit, is often associated to antibiotic resistance targeting the synthesis of ribosomal proteins 30S or 50S. Correlations between gene expression of *cyoA* and *cyoD* with spectral intensities were found to be significant (FDR, *p* < 0.05) across several peaks of the Raman spectra, including for the wavenumbers 730 and 1476 cm^−1^, as shown in Fig. 4c. These wavenumbers are associated to nucleic acids and the cytochrome *bo* subunit (Table 2), respectively. The wavenumber 730 cm^−1^ was identified as being important for classification of this group (Fig. 3). Strains associated with protein-related antibiotic resistance exhibited various gene expression and spectral intensities at the wavenumbers shown. We noted that the strain NM, which has a mutation in the *cyoA* gene, exhibited a significantly lower gene expression for *cyoD* (ANOVA, Tukey HSD, *p* < 0.05), and had significantly higher spectral intensities (ANOVA, Tukey HSD, *p* < 0.05) at these wavenumbers by comparison to the other strains.

Antibiotic resistance associated with the folic acid synthesis often involves mutations in the *folACD* operon, which encodes for a key enzyme involved in purine biosynthesis, and in the formation of tRNA (a precursor of DNA synthesis). Figure 5a shows correlations that were significant for two wavenumbers (FDR, *p* < 0.05). Strain TP, which has a mutation in this operon, displayed higher gene expression for *folA* and *folC*. It also had a higher spectral intensity for these wavenumbers by comparison to other strains (ANOVA, Tukey HSD, *p* < 0.05). Figure 5b displays the correlations for other genes involved in various antibiotic resistance, such as *ompF*, *nuo*, *dinB*, and *shd*, all of which were significant (FDR, *p* < 0.05). The CFPX strain, which has fixed mutations in *nuoA* (Table 1), showed the lowest level of gene expression for *nuoA* when compared to the other strains, and exhibited the lowest and highest spectral intensities at selected wavenumbers (Fig. 5b).

## Discussion

In the past decade, the application of vibrational spectroscopy to identify antibiotic resistance has become a promising avenue of research leading to the development of diagnostic methods^14, 16–18^. Previous studies have focused on monitoring phenotypic responses by bacteria in response to direct drug exposure to evaluate the impact of antibiotic treatment or to investigate the antibiotic mode of action^4–18^.

In this study, we demonstrated the use of Raman spectral information in identifying bacterial cell type (Fig. 1b) and the mode of action of antibiotic resistance (Fig. 2b) in the absence of antibiotic drugs in closely related strains of *E. coli*. Interestingly, two of the modes of action could not be distinguished in the two-dimensional DA–PC space (Fig. 2b), and both modes related to the inhibition of mRNA translation through binding to the ribosome (30S and 50S subunits). Moreover, to assess the generalization power of our model to reliably predict antibiotic resistance in independent experiments, we trained our model on the 416 population measured across two experiments and tested the model on a third experiment that includes 208 population (Supplementary Figure 6). The accuracy of the predictions demonstrates the ability of Raman spectroscopy to produce fine-grained, reliable, phenotypic signatures for the characterization of antibiotic resistance even though strains are closely related. Further investigations could address the antibiotic resistance dynamics in unknown genetic background or a broader range of species by performing analytical models trained on spectral database established on a large variety of species, as suggested in previous studies^16–18^.

The above results suggest that spectral signatures of bacteria cells reflect the overall metabolic response of acquired resistance. However, it remains a challenge to determine which aspects of the wavenumbers of a Raman spectrum are linked to the internal information of cells. The discriminant vectors of the DA–PC model helped in identifying the spectral regions that contributed the most in the discrimination of the different modes of action of antibiotic resistance (Fig. 3), and therefore would be the most representative of metabolic differences across the antibiotic-resistant strains of this study. We focused on the analysis of the two first components because they possess most of the discriminant information needed to distinguish the mode of actions (Fig. 2b). We envisioned that the identified groups of wavenumbers could be used to generate testable hypotheses in order to decipher the complex relationships between antibiotic resistance and the nature of Raman spectral information. We performed an exploratory approach to verify (i) if any correlation could be found between Raman spectral information and gene expression, (ii) if the spectral regions or peaks found to contribute to the classification of the mode of actions correlated with the genes possibly involved in the resistance, and (iii) discuss if there metabolic/structural link could be found between a given gene and wavelength.

We found significant correlations (|*R*| > 0.601, FDR *p* < 0.05) between Raman spectral intensities and gene expression. This result alone suggests that some metabolic or structural relationship between spectral information and gene expression may exist, as exemplified below with the case of some mutant strains. We also found that the wavenumbers contributing to the classification of the mode of action (Fig. 3) could correlate with the expression of genes involved in antibiotic resistance mechanisms. For example, the wavenumbers 950 and 1209 cm^−1^, associated to lipids and proteins, respectively, strongly correlated with the expression of the *acr* and *ela* operons, respectively (Fig. 4a, Supplementary Table 1). These operons are known to code for membrane proteins associated to multidrug efflux or resistance to oxidative stress^22, 23^. Regarding the mode of action relating to DNA gyrase, strong correlations were found between two wavenumbers associated with nucleic acids and the gene expression of the *gyr* operon, and its regulator *mipA*. The two DNA gyrase mutant strains exhibited higher gene expression and higher spectral intensities by comparison to other strains (Fig. 4b, Table 2), suggesting the chosen combinations of gene and wavenumber are representative of the mode of action of antibiotic resistance. Furthermore, the wavenumbers 752 and 1450 cm^−1^ were identified as contributing to the classification of the folic acid synthesis (Fig. 3). These two wavelengths were also found to correlate with the gene expression of the *fol* operon (Fig. 5a, Supplementary Table 1). Interestingly, the TP strain, which is related to folic acid resistance, displayed higher levels of gene expression of the *fol* operon, and higher spectral intensities at the shown wavenumbers (Fig. 5a). Thus, these wavenumbers might be particularly relevant to monitor the expression of the *fol* operon, a key compound in the tetrahydrofolate metabolic cycle.

The above results highlighted linear correlations between the variations in gene expression and variations in spectral intensities for some genes of interests. However, most likely, the spectral variations are the products of the combined effects of the expression profile of a number of genes, which may lead to both structural and metabolic changes. Therefore, determining a straightforward biochemical interpretation that explains the occurrence of linear dependencies is more challenging.

Interesting patterns were found by looking at the specific responses of the mutant strains used in this study. For example, we mentioned about the two DNA gyrase mutant strains (Fig. 4b) and the TP strain (Fig. 5a) which position on the scatterplots could confirm a biochemical or metabolic relation between these gene expressions and wavenumbers. We also found that the gene expression of *cyo*, mutated in the NM strain (Fig. 4c), was strongly correlated to the 1476 cm^−1^ peak. Interestingly, this gene operon is known to code for the cytochrome *bo* complex in bacteria, and this wavelength has been identified as a marker for histidine in the ferrous heme components in the cytochrome *bo* complex^15^. These patterns indicated that the spectral phenotypic information may reflect the structural or metabolic effects induced by the mutations. To attempt to determine if these variations in gene expression and spectral intensities were caused by the contribution of a single mutation, or the complex interaction of multiple genes, one could establish engineered mutation strains and measure how the gene expression and Raman spectra are affected. While this was not the purpose of the present study, our approach helped in identifying possible candidates to generate testable hypotheses.

In summary, our results demonstrate that acquired antibiotic resistance, and the mode of action for the resistance, can be characterized using spectral measurements in the absence of antibiotics. A systematic method was proposed to identify the spectral regions that contribute the most to each mode of action. In addition, our exploratory work relating gene expression and Raman spectral information revealed the existence of linear relations between wavelengths associated to antibiotic resistance and the expression levels of genes involved in antibiotic resistance. The relation between Raman signatures and transcriptome is an open question for future studies and should be confirmed in other cell types and using appropriate integrative approaches. In a follow-up study, we will propose the use of a linear model to integrate these layers of information which we think are complementary. To study how these two layers of information are connected through the intricate networks of the cell could enable many applications in basic and applied research.

## Materials and methods

### Cell culture and plate preparation

In a previous study, *E. coli* MDS42 strain was experimentally evolved for 3 months against 12 different antibiotic drugs at various concentrations^20^. In that work, the antibiotic tolerance of ten evolved clones was verified by conventional methods and were therefore selected for the present study along with the parental strain (Table 1). Frozen pure bacterial stocks (−80 °C) were cultured in 10 mL modified M9 medium^24^ in test tubes placed in water bath shakers with 150 strokes/min shaking (Personal-11, Taitec Co., Saitama, Japan). Cells were transferred to 96-well microplates (3595, Corning Inc., NY, USA) and cultured in modified M9 medium using an automated culture system^25^ which consists of a Biomek® NX span8 laboratory automated workstation (Beckman Coulter, Tokyo, Japan) in a clean booth connected to a microplate reader, a shaker incubator (STX44; Liconic, Mauren, LI), and a microplate hotel (LPX220, Liconic, Mauren, LI). To avoid growth phase variations, the cell cultures were synchronized in subsequent measurements by using robotic culturing. To do so, we conducted a preliminary experiment in which eight cultures with different initial cell density values (ranging from 10^−3^ to 10^−5^ of OD_600_) were prepared for each line. These culture series (88 cultures in total) were incubated and the OD values were monitored. Based on this information, we determined the initial cell density for which the growth phase of each line would reach the same point prior to the measurements by Raman spectroscopy. Prior to Raman spectroscopic analysis, cells of synchronized cultures were harvested at the beginning of the stationary phase by centrifugation at 9000*g* at room temperature for 2 min and washed with PBS buffer. Optical density was measured at 600 nm (OD_600_) by microplate reader (1420 ARVO, PerkinElmer Inc., Waltham, USA) and cells of individual population at an OD_600_ value of 1.0 (equivalent of 2.0 × 10^9^ cells) were transferred in optical glass-bottom 96-well microplates (265300, Thermo Fisher Scientific Co., Ltd., Waltham, USA). This OD value was applied to all population of all strains.

### Optical setup and measurement of bacterial cells

A homemade confocal Raman microscope was developed for this study. We used an inverted microscope (IX81, Olympus, Tokyo, Japan) equipped with a motorized stage (BIOS-L101T-S, Opto-Sigma, Tokyo, Japan) and a heated micro-chamber (Olympus, Tokyo, Japan). A 532 nm diode-pumped solid-state laser (Ventus, Laser Quantum, UK) was focused to a few micrometers above the optical glass surface through a water-immersion objective lens (NA: 1.20, UPLSAPO60×W, Olympus, Tokyo, Japan). Back-scattered Raman scattering signal was collected by the same objective lens and, detected by an electronically-cooled CCD detector (PIXIS BR400, Princeton Instruments, NJ, USA) mounted on a polychromator (MK-300, Bunko Keiki, Tokyo, Japan). The polychromator used a 1200 g mm^−1^ grating to maximize the spectral resolution of the fingerprint region (from 600 to 1700 cm^−1^). The Rayleigh scattering background was rejected by a long-wave pass edge filter and a notch filter (LPD01-532RS and NF01-532U-25, Semrock, NY, USA). An automated shutter (SHB1T, Thorlabs, NJ, USA) was placed in front of the polychromator, and the entrance-slit width was adjusted around the size of the focal spot. The spatial resolution of our system was approximately 300 nm, and spectral resolution approximately 1 cm^−1^.

For each laboratory-evolved strain, 16 bacterial population were inoculated onto optical plates. For each plate, 16 wells containing PBS were used to measure the background signal. Moreover, for each plate, 16 wells with the parental strain MDS42 were used as internal controls. The plate design is shown in Supplementary Figure 7. This setup gave the possibility to determine the reproducibility of the automated measurements within each experiment, and to perform background subtraction. Within each experiment, three plates were measured (*n* = 208 cultures and *n* = 48 wells filled with buffer). Plates were measured by Raman spectroscopy immediately after transferring the cells. Within each well, measurements were performed at five positions spaced by 100 µm intervals and forming a cross pattern. Measurements were not considered to be performed at a single-cell level, although cells were trapped in the laser, cells were alive and mobile. At each position, cells were excited for 5 s (laser power at sample 25 mW, equivalent to 100 mW μm^–^^2^). The five spectra taken for a given well were averaged to obtain the mean spectrum of that well, which is equivalent to one population. Approximately 50 min were required to scan and entire 96-well plate. Cells were kept alive in a microchamber at 37°C during analysis to avoid metabolic stress^14, 16^, rather than measuring the Raman spectra of fixed or dried bacteria cells^5, 7, 9–11^. Measurements were performed using WinSpec (Princeton, NJ, USA) synchronized to a home-made automated program developed in IGOR (IGOR Pro v6, WaveMetrics, Inc., Portland, USA).

### Influence of the growth phase on Raman spectrum

In a preliminary experiment, we noticed that the rate of growth was dramatically different among the laboratory-evolved strains. Specifically, 8–16 h were needed to reach the plateau phase (high OD value), depending on the type of strain. Therefore, we examined whether the growth phase influenced the Raman spectrum of cells, and if it hindered the accuracy of classification. Bacterial cultures at various points during the growth phase of culturing, with corresponding density values ranging from 0.1 to 1.1 (OD_600_), were collected and measured by Raman spectroscopy (Supplementary Figure 8). For each strain, six different concentrations were inoculated onto optical plates and analyzed as described above. The experiment was performed in duplicate on different days. After processing the spectral data, it was pooled prior to PCA and discriminant multivariate analysis (Supplementary Figure 8). We observed an influence of the growth phase on the Raman spectrum of cells which results are discussed in the supplemental information (Supplementary discussion).

### Raman spectral pre-processing

Raman spectra of bacterial cells were treated to remove cosmic rays, and then the spectra of cells measured at five different positions within a given well. The measurements were averaged together with the mean value recorded for each well considered one cell culture. Spectra of PBS-containing wells served as negative controls and were averaged (*n* = 16 for each plate) and used to perform background subtraction analysis. A polynomial baseline correction using the ModPoly algorithm was applied on background subtracted spectra^26^, and then the spectral data were vector-normalized. To compare Raman spectra from different experiments generated on different days, and to compensate for the imperfect reproducibility of the grating angle in the polychromator, data were interpolated using a cubic spline function. As a result, 1111 spectral variables were obtained for the range 600–1710 cm^−1^. These pre-processing steps were conducted using a homemade program in MatLab (MatLab 2015a, Mathworks, USA).

To determine if the spectral variations were significant among strains, ANOVA *F*-values and Fisher scores were performed using the null hypothesis that there were no significant differences among strains (Supplementary Figure 1, Supplementary Figure 4). Both methods calculate the ratio of inter-group variance (i.e., across strains) to intra-group variance (i.e., within each strain). A ratio of 1 shows that there is no difference between the intra-group variance and the inter-group variance. A ratio greater than 1 indicates that the inter-group variance is stronger than the intra-group variance. While Fisher score is simply defined as a ratio, the ANOVA *F*-value takes into account *F*-distribution, and therefore the *F*-test can be applied to the ANOVA *F*-values to evaluate statistical significance (*p* value).

### Multivariate analyses

For the purpose of classification, the spectral data were analyzed by PCA^27^ followed by discriminant analysis, an approach we named DA–PC. Calculations were performed using JMP software (JMP^®^, Version v11, SAS Institute Inc., Cary, USA). The PCA allowed decomposing the spectra into a linear combination of loading vectors after extracting the number of independent components. In other words, PCs quantitatively expressed the phenotypes of cells in a space of reduced dimensions. Plots of Q residuals vs. Hotelling *T*² statistics were used to identify outliers, if any, in the datasets. Only one outlier among the 624 population was removed from our analysis. Data were separated between training and test datasets as described in the result sections. The number of PCs included in the DA was determined so that only the PCs with a statistically significant approximate *F*-value (*p* < 0.001) were included in the subsequent discriminant analysis. The approximate *F*-scores were calculated following the Wilk’s Lambda and Hotelling–Lawley Trace, as defined in the JMP suite. The number of PCs for the model shown in Figs. 1b and 2b were 8 and 7, respectively. For the models shown in Supplementary Figure 1c, and Supplementary Figure 7b, 7c, 7d, 7e, models included 4 and 8 PCs, respectively. All associated loadings are shown in the supplementary figures. The DA–PC includes a priori knowledge of the groups of observations (label). The discriminant analysis assumed that each group had a multivariate normal distribution, and calculated the Mahalanobis distance, which is the distance of an observation from the mean of a group divided by the standard deviation along the direction vector. The quadratic discriminant analysis was applied to consider that the intra-group covariance matrices are not assumed equals. For a given observation, probabilities of membership in each group were calculated based on the Mahalanobis distances, and the observation was classified to the group for which the probability was the largest.

### Gene expression and correlation values

Gene expression of the parental strain and the ten evolved clones were measured in a previous study using Affymetrix microarray without any exposure to the antibiotic drug^20^. The resulting gene expression data of Affymetrix microarray are expressed in terms of quantile-normalized expression ratio. To perform scatter plots of gene expression with Raman spectral intensities, the average spectrum of each strain was calculated (*n* = 16 for evolved strains, *n* = 48 for the parental strain) for pairwise correlation with gene expression. The significance of Pearson correlation values was determined by calculating a two-tailed test on the Pearson product-moment correlation coefficient. The degree of freedom (df) was dependent on the number of independent samples. We used the averaged value of 11 strains, which gave a df of 9, and a significance threshold of 0.601 (*p* < 0.05). Adjusted *p* values were calculated using FDR correction, and values below *p* < 0.05 were considered statistically significant (Supplementary Table 1). To determine if variations in gene expression or spectral intensities were significant between mutant strains, ANOVA followed by a post-hoc Tukey HSD tests (*p* < 0.05) were performed. Calculations were conducted using JMP (JMP^®^, Version v11, SAS Institute Inc., Cary, USA).

### Data availability

Raman spectral data and gene expression datasets of the 11 strains used in this study are made available in figshare (10.6084/m9.figshare.6280796)^28^.

## Electronic supplementary material

Supplementary information
