# Supplementary material for: Raman spectral signature reflects transcriptomic features of antibiotic resistance in Escherichia coli
Source: Commun Biol. 2018 Jul 2;1:85. doi: 10.1038/s42003-018-0093-8 (PMC6123714; doi:10.1038/s42003-018-0093-8)
Supplement: Supplementary file 1 — Supplementary information [file 42003_2018_93_MOESM1_ESM.pdf]

Supplementary information

Supplementary Figures

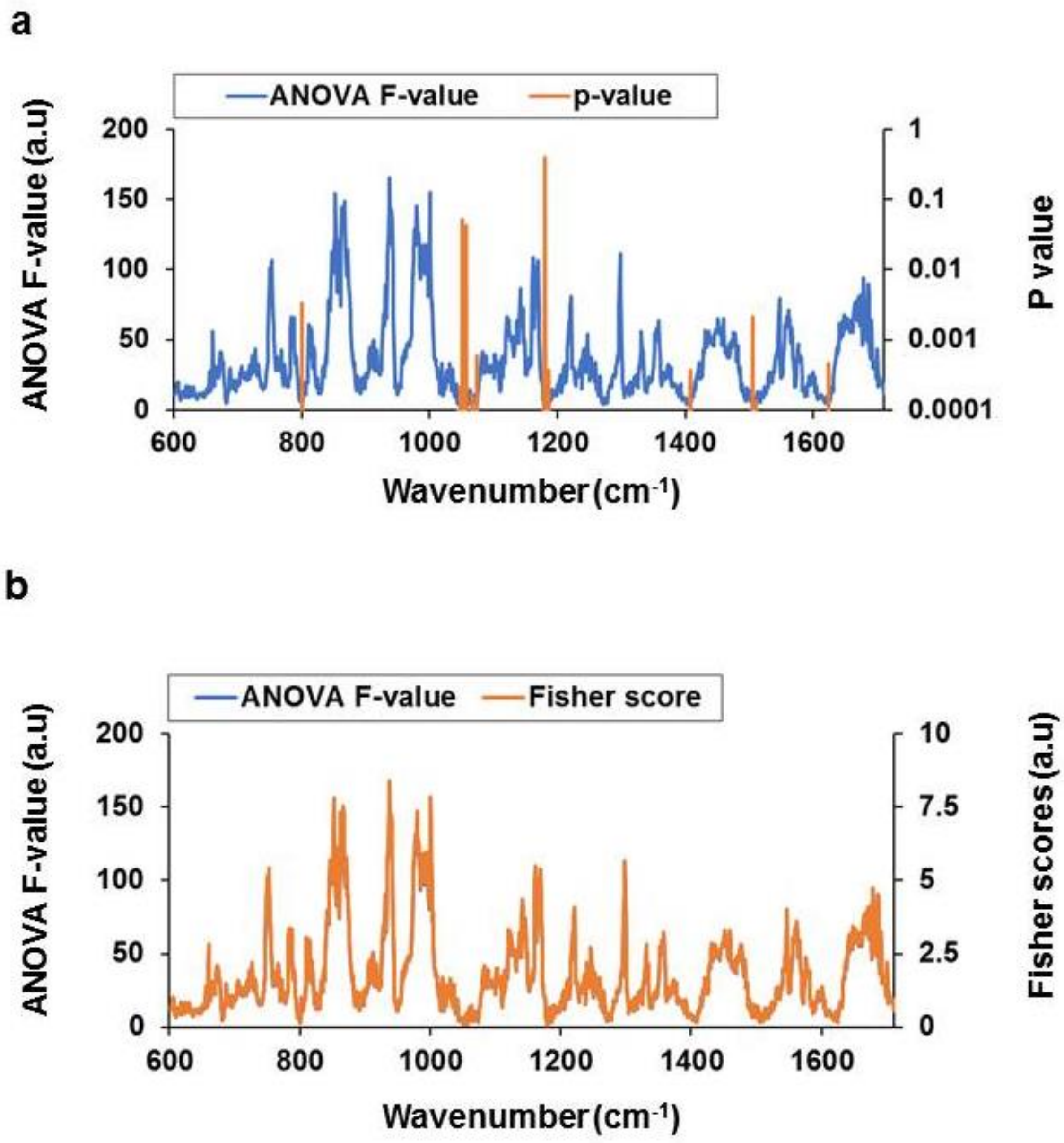

Supplementary Figure 1. Statistical significance of spectral differences between strains. (a) *F*-values calculated by ANOVA analysis and the related *p* values scores were calculated from the normalized spectral data of 208 populations across the 11 *E. coli* strains. Indicated *p* values showed that for most wavenumbers the differences were significant at $p < 0.01$. (b) Comparison of Fisher scores and ANOVA *F*-values. The spectral shapes are exactly the same, by definition. The fundamental difference between the two methods is that the ANOVA takes into account the number of samples.

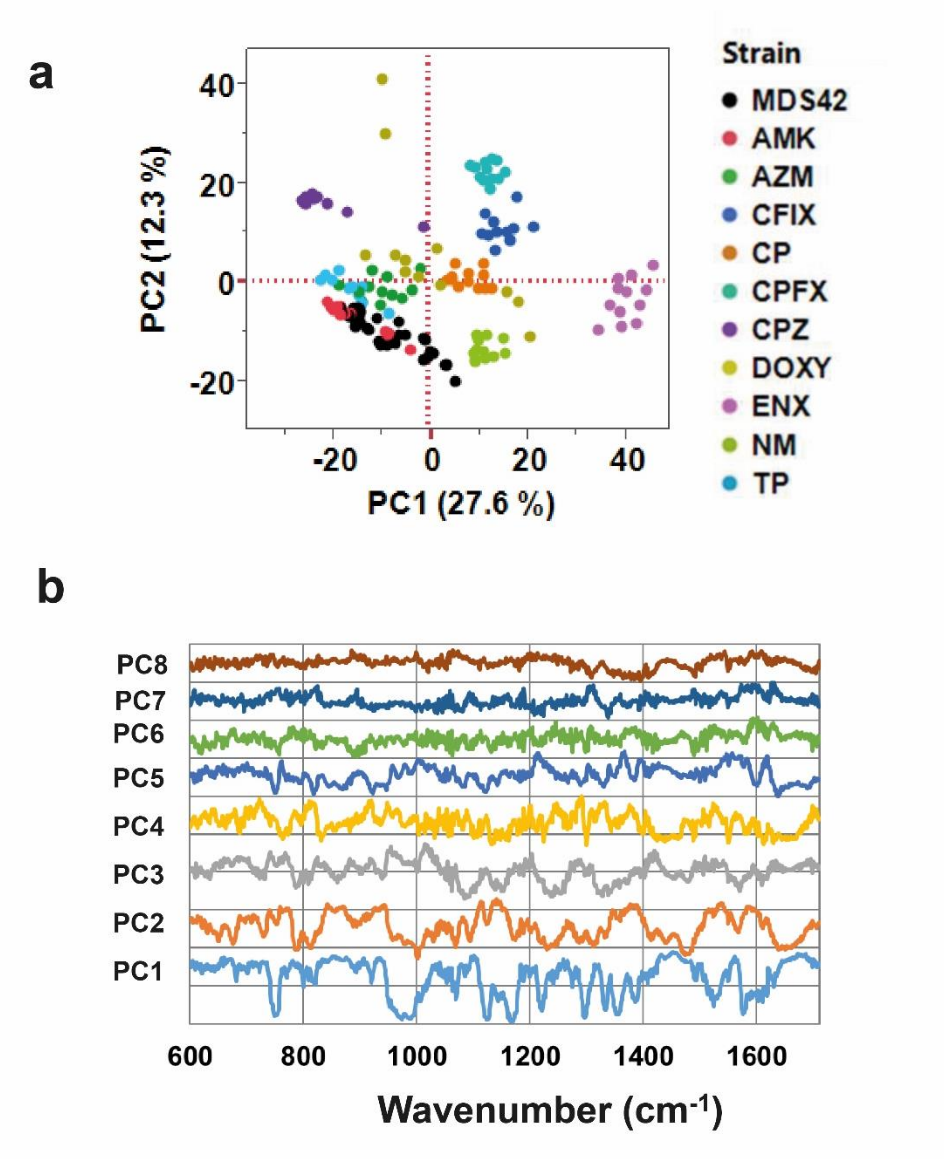

Supplementary Figure 2. PCA of spectral profiles of bacterial populations. (a) PC score plot of the spectral dataset of 208 populations across the 11 *E. coli* strains. The two-dimensional PCA space clearly indicated differences among strains. (b) Loadings vectors of the first eight PCs, which were used as input for the DA-PC model aiming at discriminating the eleven cell lines.

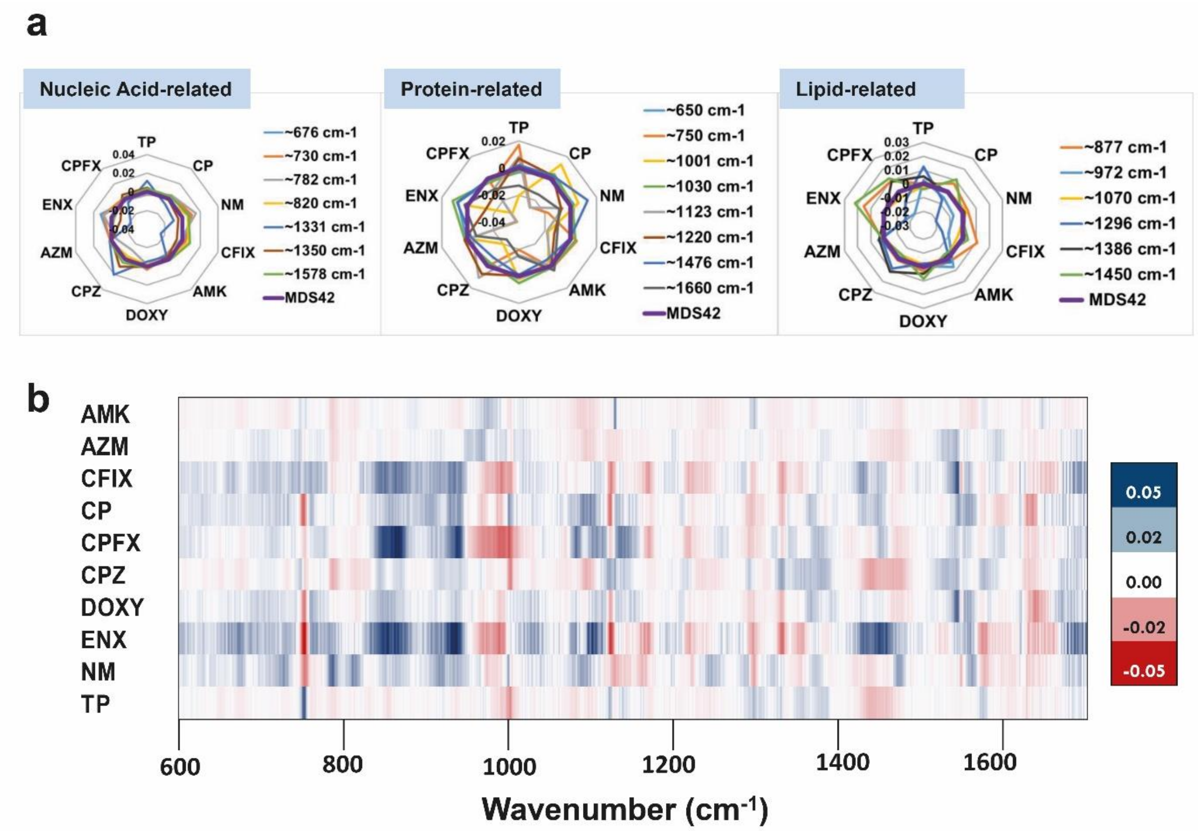

Supplementary Figure 3. Comparative analysis of relative differences in spectral intensities between the parental (control) and antibiotic resistant strains. (a) Relative differences between the maximum spectral intensities of the parental and laboratory evolved strains, for selected peaks. Peaks were categorized into three families: nucleic acid, protein, or lipid-related. The parental strain is represented by the thick violet line. (b) Colour representation of the difference spectra of each laboratory evolved strain from the parental strain, and each laboratory evolved strain over the entire spectral range. Blue represents positive differences, and red negative differences compared to the parental strain.

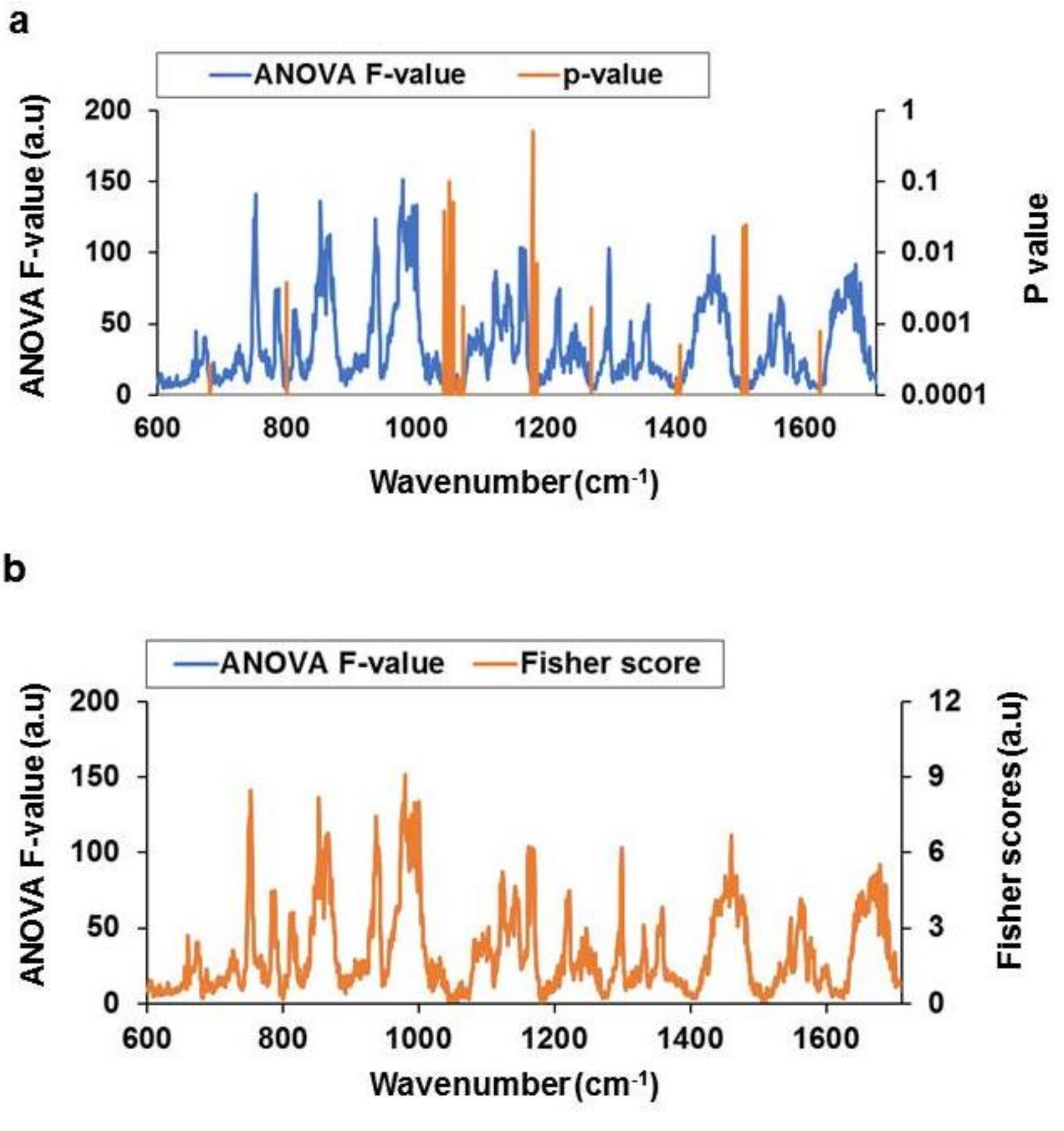

Supplementary Figure 4. Statistical significance of the relative spectral differences between the parental and laboratory evolved strains. (a) *F*-values spectrum calculated by ANOVA analysis and the associated *p* values. They were calculated from the differences between the averaged spectrum of the *E. coli* MDS42 (wild type) populations ($n = 48$) and each population of the laboratory evolved strains. Total is 160 populations. Indicated *p* values showed that except for some valley regions (e.g., 1180 cm-1) the differences were statistically significant at $p < 0.01$. (b) Comparison of Fisher scores and ANOVA *F*-values.

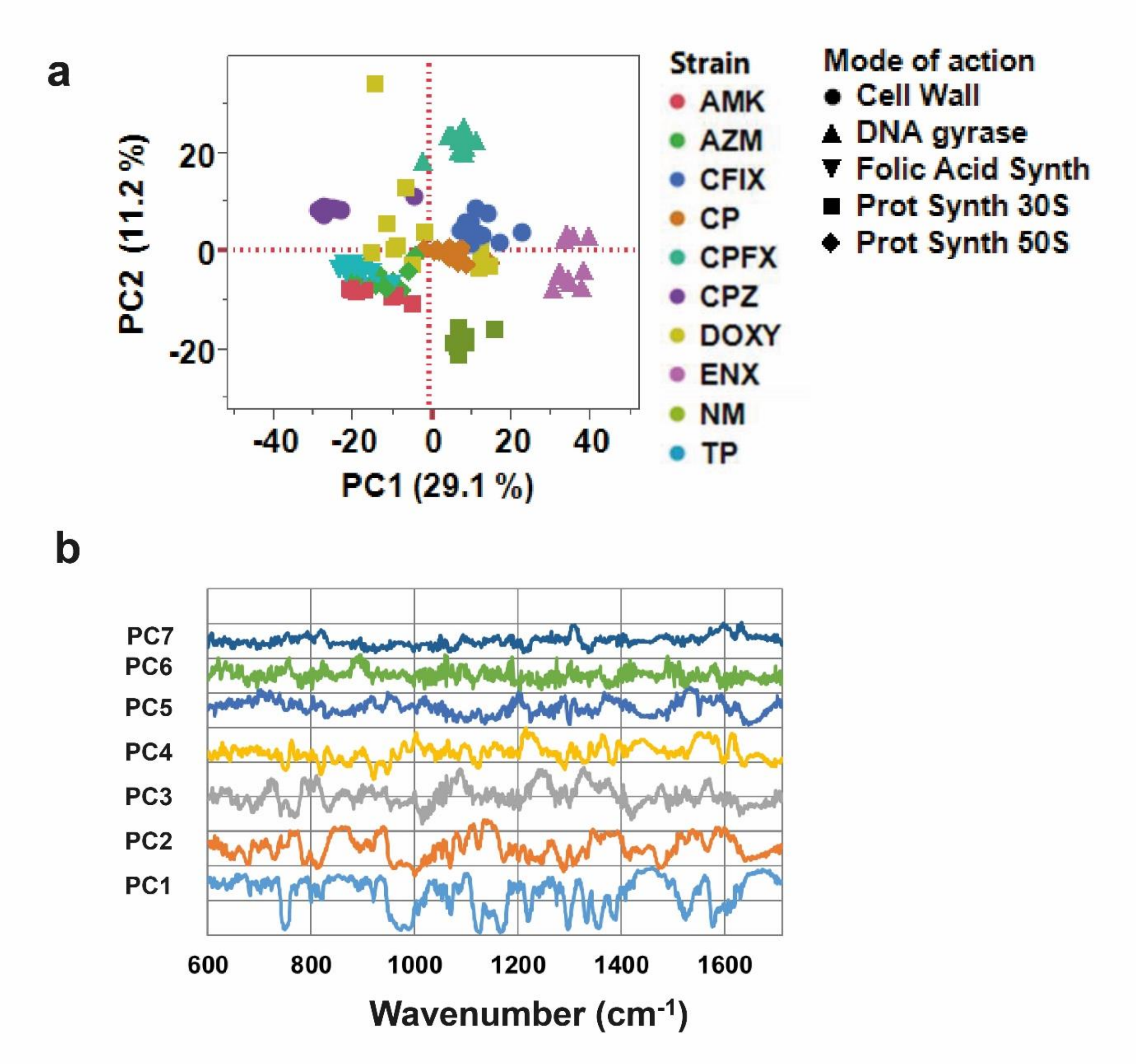

Supplementary Figure 5. PCA of the relative spectral differences between the parental and the laboratory evolved strains. (a) PC score plot for the spectral dataset of 159 populations. The two-dimensional PCA space clearly demonstrated differences among the strains. (b) Loadings vectors of the first seven PCs, which were used as input for the DA-PC model of the modes of action of antibiotic resistance.

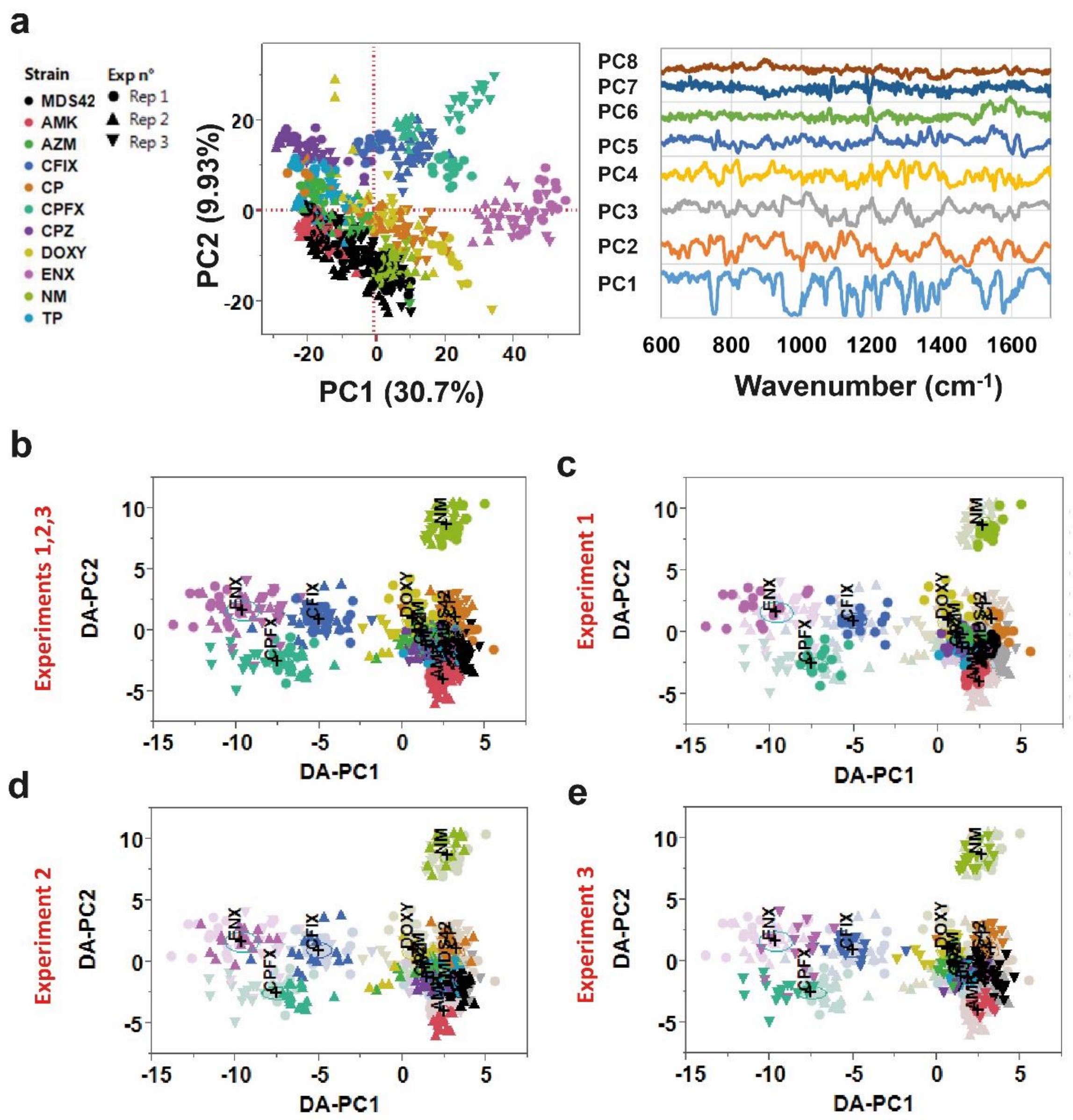

Supplementary Figure 6. Demonstration of the reproducibility of discrimination based on the spectral information obtained from three independent experiments.

(a) PC score plot of Raman spectra obtained from three independent experiments performed in different weeks. The total number of cell cultures was 624. (b) Score plot of DA-PC model built on eight PCs. The model showed that each strain occupied a specific position in the two-dimensional space. A Wilks' Lambda test (F_{value} = 152.52; $p < 0.0001$) and Hotelling-Lawley (F = 288.05; $p < 0.0001$) showed that the means of the covariates were significantly different across groups. Misclassified data accounted for 3.7% of the data in this model. These results demonstrate the reproducibility of the method to discriminate the cells based on their spectral information. (c, d, e) DA-PC score plots for each independent experiment, separately shown in the same space defined by the DA-PC model by colouring the data of the other experiments in grey. In the two-dimensional space, small intra-group variations could be observed among the three experiments. For example, the position of the NM strain varied

slightly in experiments 1, 2, and 3. These variations did not significantly hinder the ability to discriminate the strains from each other (inter-group variation).

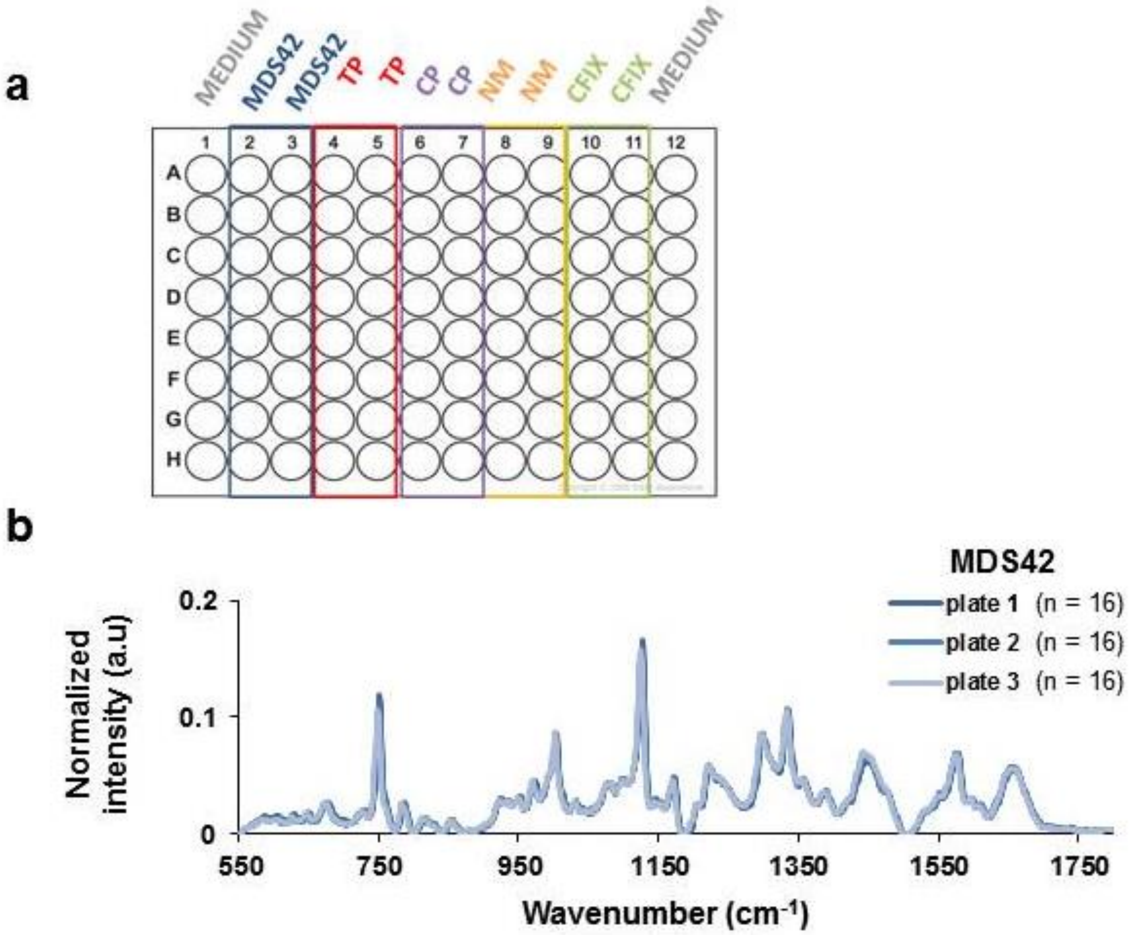

Supplementary Figure 7. Methodology for the automated measurement of Raman spectra of independent bacterial cell cultures. (a) Sample assignment of each well in one 96-well plate. For each bacteria strain, 16 cell cultures (biological replicates) of the desired optical density (OD) value were immediately transferred to optical-glass bottom 96-well plate and measured under the same temperature conditions. On each side of a plate, 8 wells were used to measure the background signal (PBS). Since we used parental *E. coli* strain MDS42 as an internal control, only four additional strains could be placed on each 96-well plate (for illustration here, laboratory evolved strains TP, CP, NM and CFIX). Therefore, three plates were used in each experiment to collect the Raman spectra of the eleven strains. (b) Reproducibility of Raman spectral measurements assessed by using parental MDS42 as an internal control on each 96-well plate. Each spectrum represents the average of 16 replicates. Small variations may be linked to small biological variation and/or sample preparation variations. However, these variations did not hinder the ability to discriminate the different strains as seen in the classification models.

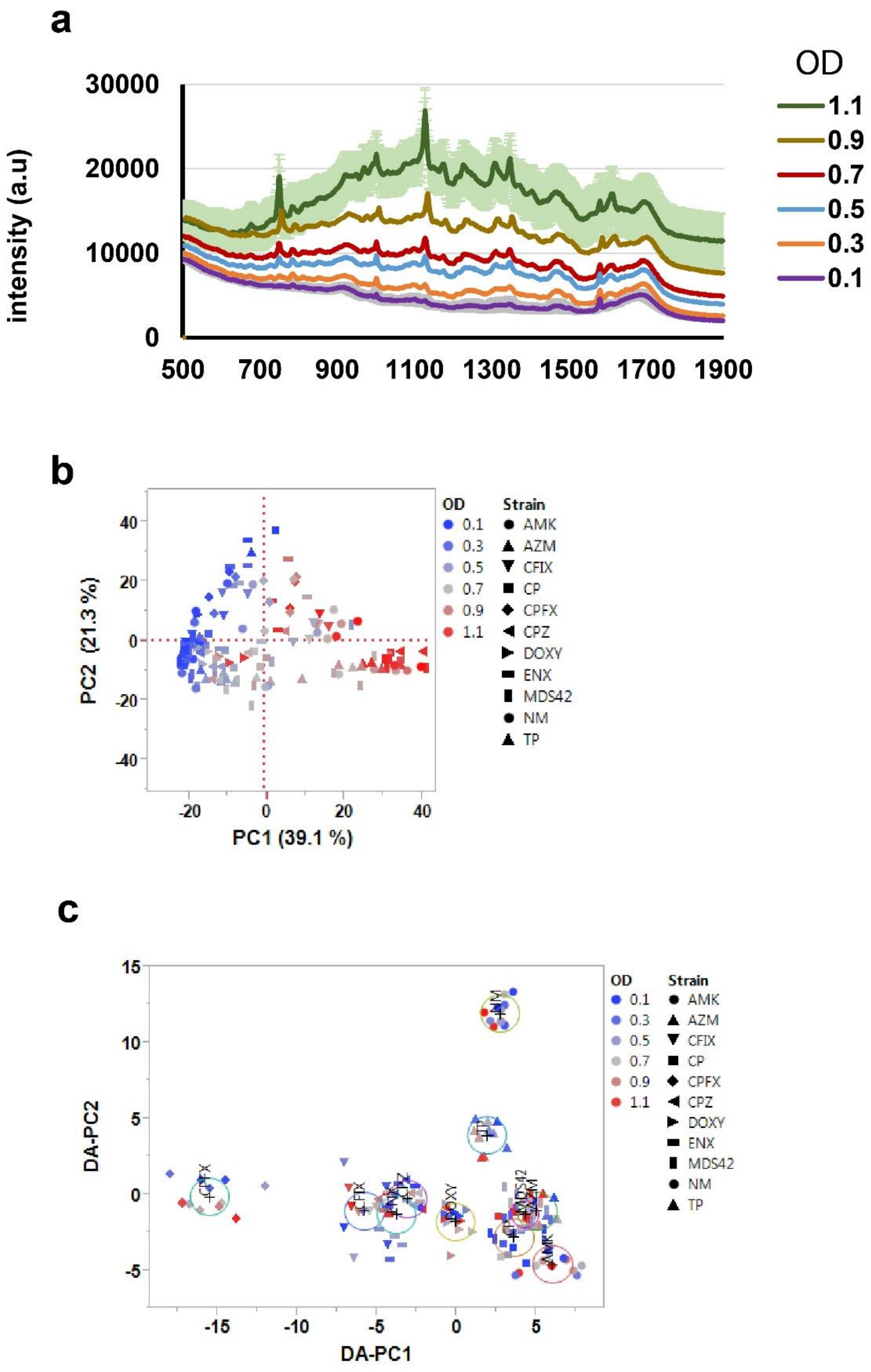

Supplementary Figure 8. Evaluation of the effect of growth phase on the classification of bacterial strains. (a) Raw spectral data of bacterial culture of the TP strain measured five time each in independent well at different OD_{600} values. Lines represent average spectral data across 5 measurements. Highlighted areas corresponding to the standard deviation of spectral measurement across five population. To improve the clarity of the figure, these areas are shown for only two OD_{600} values, at 1.1 and 0.1 OD. The raw spectral data show that the relative contribution of cytochrome peaks increases with increasing OD values. (b) PCA

score plot of the eleven *E. coli* strains at various growth phase (n = 144). Each point on the graphic corresponds to one cell-culture (i.e., biological replicate). Two dimensional PCA indicated some contribution of the OD value on the spectral measures. This result supported that variations of the growth rate influence the Raman spectra as reported in previous studies[1-3]. (c) DA-PC model to classify populations by strain. Each population was coloured according to its OD_{600} value, and different marker shapes indicate different strains. The results demonstrate that the strains could be classified by type, despite different OD_{600} values. The classification error rate was 15.9%. This high error rate suggests that the variations of growth phase partially hindered the ability to discriminate the strains accurately from each other. This finding justified our choice to use synchronized cultures in subsequent experiments in order to avoid this effect (see Material and Methods).

Supplementary Tables

Supplementary Table 1. Correlation and associated *p* values for tested pairs of genes and spectral wavenumbers. Pearson correlations and associated *p* values are shown along with the adjusted *p* values calculated using FDR correction. Values lower than $p < 0.05$ were considered as significant.

Gene	Peak (cm^{-1})	Correlation R value	*p* value	Adjusted *p* value (FDR)
acrA	950	-0.69	0.0181	0.0377
acrB	950	-0.67	0.0243	0.0379
elaA	1209	0.63	0.0360	0.0425
elaA	1545	0.49	0.1263	0.1263
elaB	1209	0.92	0.0001	0.0017
elaB	1545	0.76	0.0072	0.0277
cyoA	730	-0.85	0.0010	0.0102
cyoA	1476	-0.75	0.0076	0.0277
cyoD	730	-0.85	0.0010	0.0102
cyoD	1476	-0.76	0.0069	0.0277
gyrA	1079	0.63	0.0395	0.0433
gyrA	1101	0.63	0.0367	0.0425
gyrB	1079	0.67	0.0249	0.0380
gyrB	1101	0.62	0.0403	0.0433
mipA	1079	-0.65	0.0315	0.0415
mipA	1101	-0.69	0.0195	0.0377
folA	752	0.69	0.0182	0.0377
folA	1450	-0.61	0.0480	0.0498
folC	752	0.78	0.0045	0.0263
folC	1450	-0.73	0.0103	0.0291
ompF	853	-0.72	0.0133	0.0321
ompF	936	-0.68	0.0221	0.0380
ompF	1070	0.83	0.0016	0.0116

nuoA	1030	0.67	0.0228	0.0380
nuoA	1079	-0.74	0.0095	0.0291
dinB	752	-0.73	0.0110	0.0291
dinB	1101	0.64	0.0331	0.0417
sdhA	752	0.65	0.0314	0.0415
sdhA	1101	-0.66	0.0286	0.0414

Supplementary discussion

Influence of growth phase on the raw Raman spectra

It has been previously reported that the growth phase can influence the Raman spectrum of single bacterial cells [1,2]. We conducted a preliminary analysis to verify how different OD values could affect the Raman spectra, and the subsequent discrimination of cell lines. Bacterial populations of eleven strains were collected for six different OD_{600} values and placed in 96-well plates for Raman spectroscopy measurements. Cells were measured at five different locations and averaged to produce the average spectrum of a given population (Materials and Methods). The Supplementary Figure 8a show the raw averaged spectra of the TP strain, which strain was selected randomly. The spectra clearly show that the peak intensities increase with higher OD values.

The cytochrome and proteins peaks were particularly dominant in the spectra of the populations cultured to higher OD values. The high intensities of the peaks at 750 cm^{-1} and 1130 cm^{-1}, both associated to cytochrome, are most likely linked to the higher cytochrome activity occurring at higher OD, as suggested by previous publication [3]. During the review process, we were asked to do a comparison with previous studies, and we thought this information could be of interest. Moritz and colleagues also tested the influence of OD on the spectral signal, and no increase of cytochrome peaks was visible with increasing OD values [10]. This is likely due to the spectral processing applied by the authors, who normalized the spectral intensities against peak intensities at 4 hours after inoculation of the bacteria. The intensity of the signal strongly depends on the peak intensity chosen for normalization process, which information is not given. Moreover, this study used a different excitation wavelength (785 nm), which may partially account for differences.

In our main experiment, cells were collected at the end of log phase (OD=1), for which the cytochrome activity could be the strongest. In other studies, OD was adjusted to 0.3 or 0.4 [4], or to 0.5 [5]. Moreover, one of these studies (i.e., [5]) dried the cell suspensions prior to analysis. Based on our experience, drying and or fixation of cells causes a strong degradation of cytochrome and RNA-related peaks.

Influence of growth phase on the statistical discrimination of strains

Then, we examined if the growth phase undermined the discrimination of the bacterial strains. After proper background subtraction and normalization of the above raw spectral data, multivariate analyses were used to evaluate the effect of growth phases on the classification. Experiments were performed twice, and their results pooled (Supplementary Figure 8b). Comparison of the raw data between the various states of growth revealed differences in the intensities of peaks related to cytochrome, nucleic acids, and proteins. To visualize the spectral difference in a space of reduced dimensions, PCA was applied on the averaged spectrum of each population (Supplementary Figure 8b). The PCA model

confirmed the influence of growth phase on classification, as seen by the pattern of distribution between bacteria of different OD values. A DA-PC model built on the PCs components (Supplementary Figure 8c) successfully discriminated the different strains with a classification error rate of 15.9%. This misclassification error was higher than compared to results shown in Fig. 1. This suggests that the variations in the growth phase reduced the ability to discriminate the strains from each other, although it did not completely impede the discriminative power of the model. Therefore, for our main analysis, we chose to use take advantage of robotic culture to synchronize our culture, and compare bacterial populations obtained at one specific state of the growth cycle (see Material and methods). This ensured that our experimental results were not biased from the variations in growth-state.

Supplementary references

1. Moritz, T. J. *et al.* Evaluation of Escherichia coli cell response to antibiotic treatment by use of Raman spectroscopy with laser tweezers. *J. Clin. Microbiol.* **48,** 4287–4290 (2010).
2. Xie, C. *et al.* Identification of single bacterial cells in aqueous solution using confocal laser tweezers Raman spectroscopy. *Anal. Chem.* **77,** 4390–4397 (2005).
3. Faller, A. H. & Schleifer, K.-H. Effects of growth phase and oxygen supply on the cytochrome composition and morphology of Arthrobacter crystallopoietes. *Curr. Microbiol.* **6,** 253–258 (1981).
4. Stöckel, S., Kirchhoff, J., Neugebauer, U., Rösch, P. & Popp, J. The application of Raman spectroscopy for the detection and identification of microorganisms: Raman spectroscopy for microorganism detection and identification. *J. Raman Spectrosc.* **47,** 89–109 (2016).
5. Athamneh, A. I. M., Alajlouni, R. A., Wallace, R. S., Seleem, M. N. & Senger, R. S. Phenotypic profiling of antibiotic response signatures in Escherichia coli using Raman spectroscopy. *Antimicrob. Agents Chemother.* **58,** 1302–1314 (2014).
